# Effect of prone positioning in adult patients receiving veno-venous extracorporeal membrane oxygenation: A meta-analysis

**DOI:** 10.1371/journal.pone.0320532

**Published:** 2025-03-25

**Authors:** Dehua Zeng, Aiqun Zhu, Jiayi Zhao

**Affiliations:** 1 Xiangya Nursing School of Central South University, Changsha, Hunan, China; 2 Clinical Nursing Teaching and Research Section, The Second Xiangya Hospital of Central South University, Changsha, Hunan, China; 3 Department of Emergency Medicine, The Second Xiangya Hospital of Central South University, Changsha, Hunan, China; Stamford Health System: Stamford Hospital, UNITED STATES OF AMERICA

## Abstract

**Objective:**

To investigate the effects of prone positioning during extracorporeal membrane oxygenation (ECMO) and its effects on short-term and long-term survival.

**Methods:**

A computerized search was performed for all studies in PubMed, Web of Science, Embase, and the Cochrane Library up to December 31, 2023, including prospective and retrospective clinical studies of ECMO-treated patients with or without prone positioning. Titles, abstracts, and full-text articles were screened in duplicate by two investigators. The primary outcome was short‐term survival (survival at discharge or 1-month survival). The secondary outcomes included long-term survival (60-day survival, 90-day survival), ECMO duration, length of intensive care unit (ICU) stay and ECMO weaning.

**Results:**

Fifteen studies with 2608 patients were included, most of which were retrospective. The effect of prone versus non-prone positioning in ECMO patients was OR =  1.32; 95% CI, 0.88–1.97; P =  0.18 for short-term survival from the original data. The effects of prone positioning during ECMO were a significant increase in 28-day survival (OR = 2.54; 95% CI 1.71–3.76; P < 0.00001) and survival at discharge (OR = 1.49; 95% CI 1.11–2.00; P = 0.009), which appeared in the non-COVID-19 patient group. Furthermore, the short-term effects of prone ventilation in ECMO patients were also improved in the matching analysis (OR = 1.66; 95% CI, 1.23–2.23; P = 0.0008), but did not in the long-term survival rate (OR = 1.57; 95% CI, 0.90–2.76; P = 0.11). The durations of ECMO (OR = 1.99; 95% CI, 1.99–2.70; P < 0.00001) and ICU stay (OR = 1.17; 95% CI, 0.58–1.75; P < 0.0001) were significantly different between the prone group and the non-prone group.

**Conclusion:**

Prone position ventilation during ECMO confers no significant advantage in improving long-term survival and only slightly benefits short-term survival. Therefore, the prone position during ECMO should be carefully considered because further randomized clinical trials on this subject are needed.

## 1. Introduction

Extracorporeal membrane oxygenation (ECMO), also known as extracorporeal life support (ECLS), is an extracorporeal technique that provides prolonged cardiac and respiratory support to patients whose heart and lungs cannot provide sufficient gas exchange or perfusion to sustain life. There are two main ECMO modes, namely, veno-venous ECMO (VV-ECMO) and veno-arterial ECMO (VA-ECMO). Currently, VV-ECMO is used for refractory severe hypoxemia or respiratory failure when conventional treatments fail, such as in patients with severe pneumonia and severe acute respiratory distress syndrome (ARDS) [1]. VA-ECMO provides cardiopulmonary support, which is typically used for fatal cardiogenic shock and cardiac arrest [2]. Acute respiratory distress syndrome (ARDS) is an acute diffuse inflammatory lung injury that can lead to increased pulmonary vascular permeability, increased lung weight, and decreased lung tissue ventilation [3]. Studies have shown that patients with acute respiratory distress syndrome account for approximately 10.4% of intensive care unit (ICU) hospitalizations, and the mortality of severe ARDS hospitalizations can reach 46.1% [4]. When traditional mechanical ventilation, lung-protective ventilation strategies, and prone positioning do not improve hypoxia in patients, VV ECMO is the preferred salvage treatment for patients with ARDS [4]. With the development of scientific research, improvements in technology, and the lower cost of consumables, the use of ECMO, especially VV-ECMO, is rapidly increasing worldwide [5].

The physiological benefits of prone position (PP) ventilation include increasing functional residual air volume, promoting alveolar expansion, improving the ventilation/perfusion ratio, altering diaphragmatic motion, decreasing cardiac compression, and improving hemodynamics [6]. In early studies, the prone position was proposed for patients with acute hypoxemic respiratory failure [4,5], moderate to severe ARDS [6], and COVID-19 acute respiratory failure [7], and the results revealed that the prone position significantly improved oxygenation and reduced intubation rates, ICU admission rates, and mortality. Once a patient’s hypoxia is refractory to the prone position, ECMO is one of the auxiliary and salvage treatment measures. Some patients undergoing prone ventilation while receiving ECMO support show significant clinical effects compared with non-prone ventilation patients, with improved oxygenation, respiratory system compliance, and no serious adverse events [7–9]. With further research and implementation, the effectiveness of ECMO has received the attention of a large number of ICU specialists and has been recommended by relevant guidelines [10]: prone position ventilation does not confer significant advantages in the short-term and long-term effects of ARDS, and only its use in moderate to severe ARDS has been suggested. The guidelines also do not mention the prone position for VV-ECMO patients. Therefore, it is necessary to evaluate the effectiveness of the prone position during ECMO.

To what extent will the physiological benefits of implementing prone ventilation on the basis of ECMO be obtained for critically ill patients? The use of supra-protective ventilation (i.e., 3–4 mL/kg tidal volume) during ECMO increases the proportion of gravity-dependent lung regions that are poorly ventilated, whereas the prone position improves oxygenation by reopening the dorsal lung zones and promoting pulmonary drainage. However, some retrospective studies have reported that ECMO combined with prone ventilation does not improve patient prognosis, such as weaning and survival [11]. A study from a multicenter randomized clinical trial also revealed that, compared with supine positioning, prone positioning did not significantly reduce the time to successful weaning of ECMO or improve clinical outcomes among patients with severe ARDS supported by VV-ECMO [12]. It cannot be denied that there are certain obstacles to the implementation of ECMO combined with prone ventilation. In addition to the lack of experience, prone positioning also requires significant equipment and training. Although intensivists are aware that prone positioning is recommended, many clinicians cannot provide this treatment modality for their patients. These studies were primarily retrospective, small-sample studies with inconsistent results. Although some reviews on this topic have been published, such as Papazian’s meta-analysis [7], the literature and data included were relatively limited, including several articles in which the researchers were contacted to obtain additional results. For patients, ECMO combined with prone ventilation may present more risks, such as the increased risks of pressure ulcers and prolonged staging. Whether ECMO patients can gain additional benefits from prone ventilation has become a current focus of debate [13]. Therefore, it is necessary to further clarify the clinical efficacy of ECMO combined with prone ventilation by increasing the sample size and analyzing multiple clinical outcomes [7].

## 2. Methods

### 2.1. Search strategy and selection criteria

The PubMed, Embase, Cochrane Library, and Web of Science databases were systematically searched, and all the articles published from database inception to December 31, 2023, were searched without language restrictions. We searched for cohort studies, case-control observational studies (prospective or retrospective), and randomized controlled trials. The inclusion criteria for patients were as follows: prone position or not during ECMO within the same study population; and the reasons for ECMO treatment, including ARDS and COVID-19, were not limited. The exclusion criteria were as follows: studies in which the investigators were unable to extract data quantitatively; studies that did not report patients receiving ECMO in the supine position; studies that did not report patient deaths and survival; studies in which the subject was defined as an infant or child; and studies with samples of fewer than 10 patients.

Different combinations of key words and random words were chosen on the basis of different databases: “prone position” or “prone ventilation” or “ventilation”; “extracorporeal membrane lung oxygenation” or “ECMO” or “extracorporeal oxygenation” (S1 Table in S2 File). Duplicate, animal, and nonoriginal studies were excluded. In addition, reference lists of all available review articles and primary studies were searched to identify studies not found in the online searches.

### 2.2. Data extraction

All literature was imported into Zotero software for further analysis. The PRISMA 2020 checklist was used for methodological evaluation in the meta-analysis and is listed in S2 Table in S2 File [14]. We did not register this review prospectively in PROSPERO.

First, we classified and created tags for the studies from different databases. Two researchers independently scanned the titles and abstracts, reviewed the full texts of potentially relevant studies according to strict inclusion and exclusion criteria, and finally cross-checked the results of the two authors to finalize the eligible studies. Disagreements regarding the inclusion of studies were resolved through joint discussion among the three authors. Data extraction was carried out using prespecified data sheets. The extracted data included study characteristics (study duration, design, year of publication, country), patient demographic characteristics (sex, age, sample size, comorbidities), and outcomes (survival at different time points, ECMO weaning, ECMO duration, and ICU stay). For the classic results of “survival on Day 28”, the investigators contacted the corresponding authors of each study as much as possible to obtain more results.

### 2.3. Outcomes

The primary outcome was short‐term survival, which we defined as either hospital survival or 1-month survival. In the studies we included, some reported 30-day survival, while some reported 1-month survival. Therefore, we combined these two measures and collectively referred to them as “1-month survival”. The secondary outcomes included long-term survival, ECMO duration, length of ICU stay, and ECMO weaning. We defined long-term survival as survival at day 60 (60-d survival) and survival at day 90 (90-d survival). We analyzed data separately for each time point and considered the pooled results as overall long-term survival.

### 2.4. Assessment of study quality

Quality was assessed independently by two authors at the individual study level and at the outcome level, and any differences were resolved through discussion. We followed the COSMOS-E guidelines on how to assess study quality and risk of bias [15]. The Newcastle‒Ottawa Scale (NOS), which consists of eight items out of a possible nine points, with a score of seven or more considered high quality, was used to assess the quality of cohort studies [16]. The methodological quality of the randomized studies was assessed by the Cochrane RoB2 tool on the basis of the randomization process, deviations from intended interventions, missing outcome data, measurement of the outcome, and selection of the reported result [17]. For the cross-sectional studies, researchers have utilized the guidelines provided by the Agency for Healthcare Research and Quality (AHRQ) to evaluate the methodological rigor [18].

The GRADE (Grading of Recommendations Assessment, Development, and Evaluation) approach was adopted to evaluate the overall quality of evidence regarding each outcome [19]. This tool defines four levels of certainty (namely, high, moderate, low, and very low), which are determined by factors such as the design of the included studies, the risk of bias, inconsistencies in the results, the indirectness of the evidence, imprecise results, publication bias, large effects, dose‒response relationships, and all plausible confounding factors and biases.

### 2.5. Statistical analysis

Statistical analyses were performed using RevMan 5.4 and Stata 17.0 software, and weighted mean differences (WMDs) and odds ratios (ORs) were used to compare continuous and dichotomous variables, respectively. All the results are reported with 95% confidence intervals (CIs). I-square (I^2^) tests were performed to assess the effect of study heterogeneity on the results of the meta-analysis. In accordance with the Cochrane review guidelines, a random-effects model was selected if there was significant heterogeneity at I^2^ >  50%; otherwise, a fixed-effects model was used.

The Cochrane Handbook mentions that when assessing publication bias, the use of a combination of methods is recommended, as no single method is completely accurate, especially if the number of studies is limited [19]. Funnel plots are used for subjective visualization, whereas Begg’s test and Egger’s test provide objective statistical tests for the presence of publication bias and have greater power [20]. Therefore, we combined Egger’s test, Begg plots, and funnel plots to improve the accuracy of the assessment of publication bias. We also performed sensitivity analysis to explore the stability and reliability of the results. The employed method serially excluded each study to determine the implications of individual studies for the pooled estimates [21]. P <  0.05 was deemed statistically significant and reported as 2-tailed.

In addition, we performed a comparative analysis of the matched data from 6 studies [11,22–26]. To minimize the effect of potential confounders, 5 studies used a 1:1 propensity score matching (PSM) method between the prone and supine groups. To reduce the risk of bias, one study conducted a matched cohort study on the basis of sex (male or female), age + /- 5 years, SOFA + /- 3 points, duration before mechanical ventilation + /- 2 days, and prone position before ECMO (yes or no).

## 3. Results

### 3.1. Study characteristics and quality assessment

**Fig 1:**
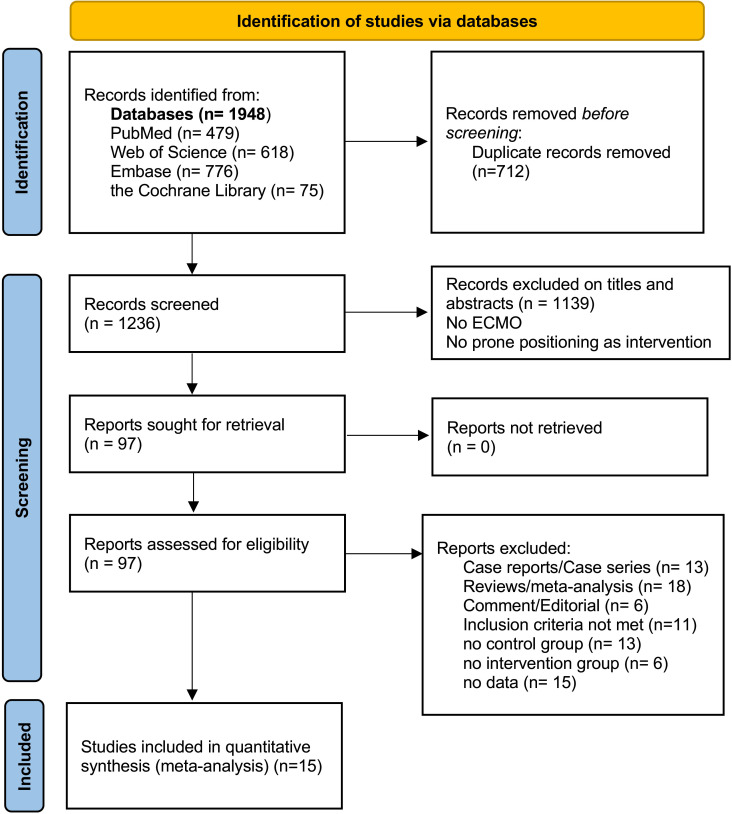
PRISMA flow diagram of study selection.

A total of 1236 publications were retrieved from the database, 97 of which were selected for full-text reading (Fig 1). The 81 studies excluded at the full-text screening stage were tabulated alongside the reason for exclusion in accordance with best practice guidelines [15,27]. A total of 2608 patients met the inclusion criteria for the final 15 studies, including 12 observational studies, one cross-sectional study, two randomized controlled trials (RCTs), and 7 articles from multicenter studies. Patients were treated with VV-ECMO in 14 of 15 studies. Another article included 11 initial VA-ECMO cases among 302 samples, followed by 5 cases that were converted to VV-ECMO, with little impact on the overall VV-ECMO results [28]. There were 6 articles on COVID-19, including 1163 (44.6%) cases [26,28–32]. Six of these studies were matched studies, including a total of 740 patients, in which ECMO patients without prone position use were similar to those with prone position use at baseline in terms of age, disease severity score, and several other clinical variables [11,22–26]. All the studies were published since 2018. The characteristics and outcomes of these studies are displayed in S3 Table in S2 File.

S4 Table S2 File shows the quality assessment of the included studies. After evaluation using the RoB2 tool, Schmidt et al’s study, a RCT, was found to have a high risk of bias. Two observational study was rated as NOS 9 stars, eight observational studies were rated as NOS 8 stars, and five were rated as NOS 7 stars. A cross-sectional study was of moderate quality/AHRQ grade. The GRADE assessment of the certainty of the evidence supporting the association of PP with better outcomes was low or very low, mainly because most studies are observational studies. The detailed quality evaluation results are listed in S4 Table in S2 File.

### 3.2. Relationship between PP and short-term survival during ECMO

**Fig 2:**
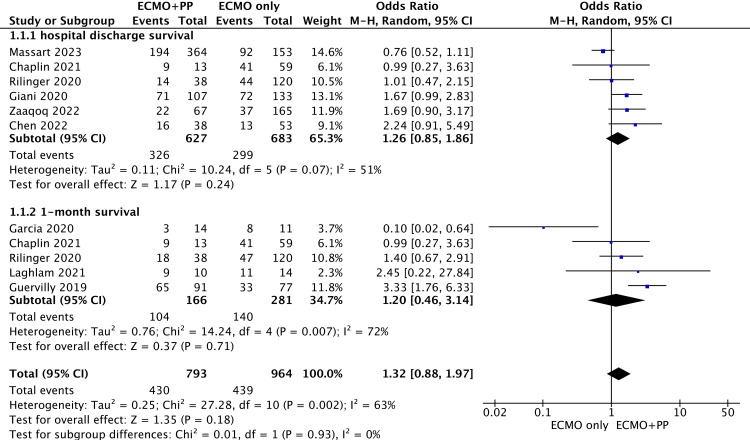
Forest plot of short-term survival.

We divided short-term survival into two subgroups for analysis (survival at discharge and survival at 1 month), as shown in Fig 2. Among the 15 studies, six [11,22,25,26,30,33] reported survival to hospital discharge, and five reported 1-month survival [11,23,29,31,33]. The pooled meta-analysis revealed no significant differences in hospital discharge survival (OR =  1.26; 95% CI, 0.85–1.86; P = 0.24) or 1-month survival (OR = 1.20; 95% CI, 0.46–3.14; P = 0.71). Pooled analysis revealed that the use of prone ventilation during ECMO was not associated with changes in short-term patient survival (OR =  1.32; 95% CI, 0.88–1.97; P =  0.18).

**Fig 3:**
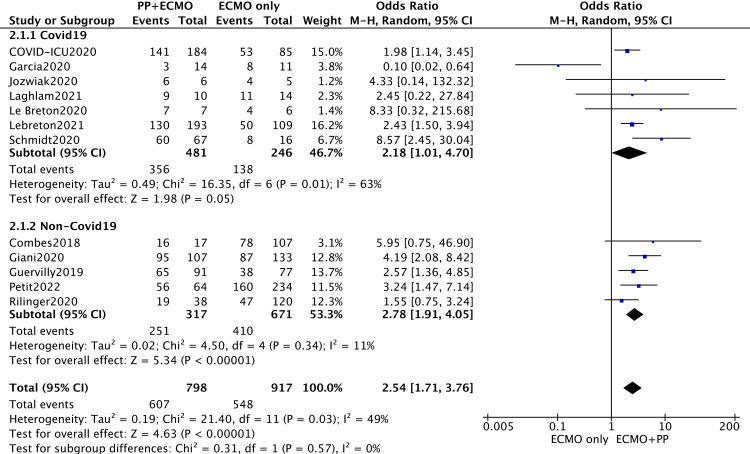
Survival on day-28 for subgroups based on COVID-19 or not.

**Fig 4:**
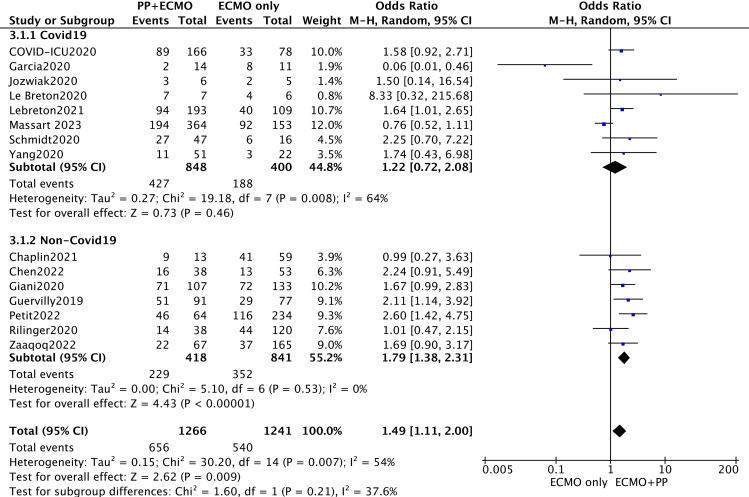
Survival at hospital discharge for subgroups based on COVID-19 or not.

The 28-day all-cause mortality rate is also a classic indicator for evaluating clinical efficacy; therefore, this study further evaluated the 28-day survival (28-d survival) rate. The outcome of 28-d survival was derived from two reports [29,31], a previous review [7], and responses to author contact. A total of 12 articles were included, and the pooled meta-analysis revealed that prone ventilation during ECMO was associated with increased 28-d survival (OR = 2.54; 95% CI 1.71–3.76; P < 0.00001) (Fig 3), and this difference was detected in the non-COVID-19 ECMO group (OR = 2.78; 95% CI 1.91–4.05). According to the same method, the pooled meta-analysis revealed that prone ventilation during ECMO was beneficial for survival at hospital discharge (OR = 1.49; 95% CI 1.11–2.00; P = 0.009) (Fig 4), and this difference was also apparent in the non-COVID-19 ECMO group (OR = 1.79; 95% CI 1.38–2.31).

### 3.3. Relationship between PP and long-term survival during ECMO

Among the 15 studies, five [12,23,29,32,33] reported 60-day survival, and five [12,23,24,28] reported 90-day survival. The pooled analysis revealed that the use of prone ventilation during ECMO was not associated with a change in 60-day survival (OR = 1.36; 95% CI, 0.78–2.37; P = 0.28) or 90-day survival (OR = 1.68; 95% CI, 1.00–2.80; P = 0.05). However, the use of prone ventilation during ECMO caused an increase in long-term survival (OR = 1.54; 95% CI, 1.08–2.21; P = 0.02) (S1 Fig in S3 File).

Furthermore, on the basis of these reported studies [23,24,28,29,32–35], a previous review [7], and responses to contacting the authors, the pooled meta-analysis revealed that prone ventilation during ECMO was significantly associated with an increase in 60-d survival (OR = 1.55; 95% CI 1.08–2.23; P = 0.02) and that there was no significant difference in 90-d survival (OR = 1.49; 95% CI 1.00–2.24; P = 0.05). There was no difference between the COVID-19 ECMO group and the non-COVID-19 ECMO group (S2 Fig, S3 Fig in S3 File).

### 3.4. Relationship between PP and short-term survival during ECMO after matching

**Fig 5:**
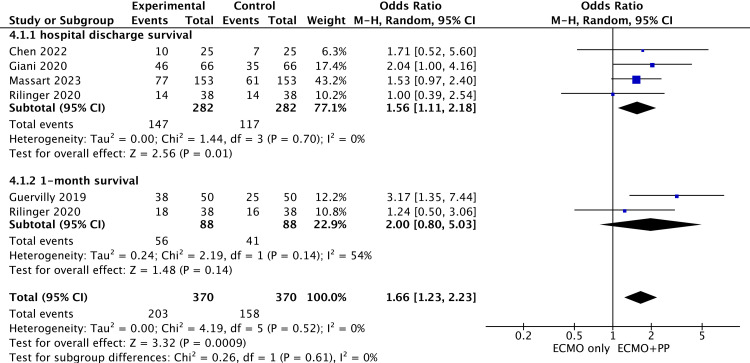
Short-term survival after matching.

We also analyzed short-term survival after matching into two subgroups (survival at hospital discharge and 1-month survival), as shown in Fig 5. Among the 15 studies, four [11,22,25,26] reported survival at discharge, and two [11,23] reported outcomes for 1-month survival. The results revealed a statistically significant difference in survival at hospital discharge (OR = 1.56; 95% CI, 1.11–2.18; P = 0.01) and no statistically significant difference in 1-month survival (OR = 2.00; 95% CI, 0.80–5.03; P = 0.14). The total effect size of the short-term survival rate after matching was OR = 1.66; 95% CI, 1.23–2.23; P = 0.0009.

### 3.5. Relationship between prone position and long-term survival during ECMO after matching

In the long-term survival matching study, three studies reported 90-day survival rates [12,23,24], and two studies reported 60-day survival rates [23,24]. The pooled analysis revealed that the use of prone ventilation during ECMO was not associated with increased 90-day survival, as shown in S4 Fig in S3 File (OR = 1.76; 95% CI, 0.79–3.91; P = 0.17). Similar results were obtained for 60-day survival in two matching studies (OR = 1.38; 95% CI, 0.47–4.10; P = 0.56).

### 3.6. Association of PP with ECMO duration, weaning rate, and ICU length of stay

ECMO duration was reported in 11 of 15 studies, and the results showed that PP during ECMO was associated with an increase in ECMO duration (OR = 1.99; 95% CI, 1.27–2.07; P < 0.00001) [11,12,22–26,29,31,33,36]. Six studies reported on ECMO weaning rates, and the results revealed that PP during ECMO was not associated with changes in ECMO weaning rates (OR = 1.17; 95% CI, 0.65–2.11; P = 0.60) [11,12,22,23,29,31]. Nine studies reported the duration of ICU stay, and the results revealed that PP during ECMO was associated with a longer duration of ICU stay (OR = 1.17; 95% CI, 0.58–1.75; P < 0.0001) (S5 Fig in S3 File) [11,12,22–26,29,36].

### 3.7. Publication bias and heterogeneity analysis

Both Begg’s test and Egger’s test were conducted to assess the publication bias of the included studies. We observed no potential publication bias with Begg’s test in S6 Fig in S3 File (hospital discharge survival: P > 1.000, 1-month survival: P = 0.462, 60-d survival: P = 0.624, 90-d survival: P = 0.806, ECMO duration: P = 0.161, ECMO weaning: P = 0.707, length of ICU stay: P = 0.348) and Egger’s test in S7 Fig in S3 File (hospital discharge survival: P = 0.330, 1-month survival: P = 0.262, 60-d survival: P = 0.903, 90-d survival: P = 0.963, ECMO duration: P = 0.384, ECMO weaning: P = 0.252, length of ICU stay: P = 0.203). The funnel plots were globally symmetrical for each outcome, although the limited number of studies did not allow the exclusion of publication bias (S8 Fig in S3 File). The P values of Egger’s and Begg’s regression intercepts were all >  0.05, suggesting that the asymmetry can be considered statistically nonsignificant.

As the subgroup data showed moderate to high heterogeneity, we performed a sensitivity analysis of the study data using Stata software using a case-by-case exclusion method, which revealed that the results were reliable and robust (S9 Fig in S3 File).

## 4. Discussion

This study reported the results of 15 published studies involving 2608 patients with and without prone positioning during ECMO therapy. Most of including studies were retrospective. The comprehensive analysis of the original data revealed that there was no significant difference in short-term survival between patients in the prone position and those not in the prone position during ECMO therapy. When referring to the previous review and responses to contacting the authors, the results revealed a significant difference in the short-term survival rate in the non-COVID-19 patient group. Furthermore, the short-term effects of prone ventilation in ECMO patients also appeared in the matching analysis. In terms of long-term survival, prone ventilation for ECMO patients does not show significant therapeutic effects.

A recent systematic review of 11 studies revealed that the cumulative survival rate (summarizing survival at the longest postdischarge time point) for patients with PP was 57%, which was not significantly greater in patients with PP than in patients without PP, and patients treated with PP had longer ICU LOS (+14.5 days, 95% CI 3.4–25.7, p =  0.01) and longer ECMO duration ( + 9.6 days, 95% CI 5.5–13.7, p <  0.0001) [37]. In a multicenter study published in The Lancet in 2023, multivariate analysis revealed that the prone position was associated with high mortality in patients treated with ECMO (HR: 1.02, 95% CI: 0.75–1.37; P = 0.916) [38]. Another review of the use of VV-ECMO for the treatment of PP in patients with ARDS revealed that the prone position was associated with significant improvement in survival at 28 days, 60 days, 90 days, and in the ICU (28-day survival: prone vs. supine group, 74% vs. 60%) [7]. In comparison, this study was based on survival at different survival time points, exploring short-term survival (1-month survival, discharge survival) and long-term survival (60- and 90-day survival), as well as length of ICU stay, duration of ECMO, and ECMO weaning rate, which showed that prone position ventilation for ECMO patients can improve short-term efficacy, especially for non-COVID-19 patients. The main benefit of PP is a more homogeneous repartition of pulmonary oxygenation stress and pulmonary oxygenation strain, which better benefits patients with hypoxemia, especially in the short term. The pulmonary changes in COVID-19 patients are primarily characterized by exudation, consolidation, and fibrosis [39]. These changes are unlikely to significantly increase functional residual capacity or promote alveolar expansion through positional changes. In fact, most ARDS patients associated with COVID-19 have more serious lung injury, and COVID-19 is related not only to lung injury but also to cardiovascular injury, immune injury, etc. In a study by Kimmoun et al, 24 hours of prone positioning was shown to improve oxygenation and respiratory compliance [9]. We recognized that there was a great deal of heterogeneity in the included studies. This may be due to the wide variation in skill levels across medical centers and the current controversies regarding the ideal populations for performing prone positioning, the start of prone positioning implementation (early OR late), and the duration of prone positioning.

Patients in the prone position had longer ECMO durations and ICU stays than did those in the non-prone position. The parameter settings of invasive ventilators are often changed during prone therapy to maintain protective or overprotective ventilation in ECMO patients, thereby allowing the lungs to ‘rest’, which may affect the judgment of the timing of ECMO weaning and easily lead to weaning delay, but this extension of ECMO duration may benefit patients: i.e., mortality is lower in patients who are under PP during ECMO [25]. Although prone position patients had longer ECMO treatment times, the weaning rate of ECMO was significantly increased, which may further prove that ECMO patients can benefit from prone position ventilation, especially critically ill patients. After matching analysis, prone position ventilation improved the short-term prognosis of ECMO patients. Another explanation is that the prone position is only indicated for patients who do not improve rapidly after ECMO; such patients may be more severely ill, and one of the reasons for ECMO initiation is that the patient is unresponsive to the prone position. Therefore, such patients require more intervention and treatment, resulting in a prolonged ECMO run-in time, which is unrelated to the prone position. A final possible reason is that the indication for the prone position in ECMO-supported patients is still unclear, and the patient’s ability to turn over in clinical practice is most likely to determine whether he or she can remain in the prone position [40].

The prone position may lead to complications such as displacement of the ECMO cannula or a sudden decrease in blood flow, which could jeopardize the patient’s life [41]. The specific incidence of each complication may vary depending on the patient’s condition and treatment conditions. There were no complications of ECMO cannula detachment during prone position treatment in the 15 included studies. Five studies reported pressure injuries [22,25,31,33,36], 9 studies reported bleeding [12,22,24,25,28,31–34], and 3 studies reported hemodynamic instability [25,33,36]. Patients who can turn over tend to be less ill, have a greater likelihood of survival, and are able to maintain prolonged interventions and ECMO therapy in the prone position with a better prognosis. Although the included studies discussed the potential benefits and risks of prone positioning, there was less specific information describing patients who were unable to be positioned prone. The decision to position patients prone was usually based on the clinician’s judgment. As far as the current clinical study is concerned, it is not possible to state separately whether the improvement in clinical prognosis was caused by the effect of prone position treatment or by the difference in the severity of the patients’ own disease between the two groups. In addition, this review is based almost exclusively on retrospective studies, where there may be many other cofactors that affect outcomes independent of prone therapy. Therefore, we look forward to more well-designed clinical trial studies with strict control of preenrollment bias and extensive follow-up in the future to further clarify the effects of prone positioning. With today’s new trends in assessing neurologic prognosis, future studies may continue to elucidate whether prone positioning during VV ECMO improves oxygenation and neurologic prognosis.

## 5. Limitations

This meta-analysis has several limitations. The main limitation was that most of the included studies were retrospective, and only two RCTs were included. Indeed, the Combes 2018 study is an RCT, but the goal of this study was not to assess the benefit of PP with ECMO, but rather the benefit of VV-ECMO during ARDS, despite the inclusion of prone and non-prone patients. Therefore, only one RCT included in this analysis addresses the benefit of PP during VV-ECMO, namely, Schmidt’s study. Second, we retrieved all VV-ECMO and VA-ECMO data, but only one study included VA-ECMO, which is far from sufficient, and more VA-ECMO research should be conducted. Methodologically, the data in this study were first extracted from the original article, followed by the authors’ replies and published reviews. We must admit that we were unable to obtain responses from all the authors or complete data. However, we also did our best to obtain the data and added new studies and content to our article. In this meta-analysis, six studies were matched to eliminate confounding factors. Owing to the influences of matching factors, sample size, and observation results, more clinical RCTs are needed to further clarify their benefits, with a focus on both short-term and long-term effects. Finally, although there was some heterogeneity in this meta-analysis, it was generally stable and reliable.

## 6. Conclusion

In conclusion, prone position ventilation during ECMO confers no significant advantage in improving long-term survival and only shows weak benefits for short-term survival when the data come from small sample matching analysis and responses from the authors. Therefore, the prone position during ECMO should be carefully considered because further randomized clinical trials are needed on this subject.

## Supporting information

S1 File Articles identified for full text assessment and all data extracted.
(XLSX)

S2 File **S1 Table.** Search equations for the different database. **S2 Table.** PRISMA 2020 checklist. **S3 Table.** Characteristics of included studies. **S4 Table.** a. Quality assessment according to Newcastle-Ottawa quality assessment scale for cohort studies. b. the Cochrane RoB2 tool for randomized controlled trial. c. Quality assessment according to Agency for Healthcare Research and Quality assessment scale for cross-sectional study. d. GRADE assess the overall quality of evidence. **S5 Table.** Criteria used for the matching procedure.
(DOCX)

S3 File **S1 Fig.** Forest plot of long-term survival. **S2 Fig.** Survival on day-60 for subgroups based on COVID-19 or not. **S3 Fig.** Survival on day-90 for subgroups based on COVID-19 or not. **S4 Fig.** Long-term survival after matching. **S5 Fig.** A ECMO duration. B ECMO weaning. C Length of ICU stay. **S6 Fig.** Publication bias was evaluated by Begg’s test: A hospital discharge survival, B 1-month survival, C 60-d survival, D 90-d survival, E ECMO duration, F ECMO weaning, G Length of ICU stay. **S7 Fig.** Publication bias was evaluated by Egger’s test: A hospital discharge survival, B 1-month survival, C 60-d survival, D 90-d survival, E ECMO duration, F ECMO weaning, G Length of ICU stay. **S8 Fig.** Publication bias was evaluated by funnel plot: A hospital discharge survival, B 1-month survival, C 60-d survival, D 90-d survival, E ECMO duration, F ECMO weaning, G Length of ICU stay. **S9 Fig.** Sensitivity analyses: A hospital discharge survival, B 1-month survival, C 60-d survival, D 90-d survival, E ECMO duration, F ECMO weaning, G Length of ICU stay.
(DOCX)
