# Supplementary material for: Effect of prone positioning in adult patients receiving veno-venous extracorporeal membrane oxygenation: A meta-analysis
Source: PLoS One. 2025 Mar 25;20(3):e0320532. doi: 10.1371/journal.pone.0320532 (PMC11936214; doi:10.1371/journal.pone.0320532)
Supplement: S2 File — S1 Table. Search equations for the different database. S2 Table. PRISMA 2020 checklist. S3 Table. Characteristics of included studies. S4 Table. a. Quality assessment according to Newcastle-Ottawa quality assessment scale for cohort studies. b. the Cochrane RoB2 tool for randomized controlled trial. c. Quality assessment according to Agency for Healthcare Research and Quality assessment scale for cross-sectional study. d. GRADE assess the overall quality of evidence. S5 Table. Criteria used for the matching procedure. (DOCX) [file pone.0320532.s002.docx]

**S1 Table. Search equations for the different database.**

| Database | Search equations |
| --- | --- |
| PubMed | ("extracorporeal membrane oxygenation"[All Fields] OR "ecmo"[All Fields] OR “extracorporeal oxygenation” [All Fields] OR "extracorporeal life support"[All Fields] OR "ecls"[All Fields] OR "membrane oxygenator"[All Fields]) AND ("prone"[All Fields] OR "prone position"[All Fields] OR "prone positioning"[All Fields] OR "proning"[All Fields] OR “prone ventilation” [All Fields]) |
| Embase | ('extracorporeal oxygenation'/exp OR 'extracorporeal membrane oxygenation'/exp OR 'ecmo'/exp OR 'extracorporeal life support'/exp OR 'ecls'/exp) AND ('prone position'/exp OR 'prone ventilation'/exp OR 'proning'/exp OR 'prone positioning'/exp OR 'prone'/exp |
| The Cochrane Library | ( (extracorporeal membrane oxygenation):ti,ab,kw OR (ECMO):ti,ab,kw OR (extracorporeal oxygenation):ti,ab,kw OR (extracorporeal life support):ti,ab,kw OR (ecls):ti,ab,kw (Word variations have been searched)) AND ((prone position):ti,ab,kw OR (prone positioning):ti,ab,kw OR (prone):ti,ab,kw OR (prone ventilation):ti,ab,kw (Word variations have been searched)) |
| Web of Science | ((TS= (extracorporeal membrane oxygenation) OR TS=(ECMO) OR TS= (extracorporeal oxygenation) OR TS= (extracorporeal life support) OR TS=(ecls) OR TS= (membrane oxygenator)) AND ((TS= (prone position) OR TS= (prone positioning) OR TS=(prone) OR TS= (prone ventilation)) |

**S2 Table. PRISMA 2020 checklist.**

| **Section and Topic** | **Item #** | **Checklist item** | **Location where item is reported** |
| --- | --- | --- | --- |
| **TITLE** | | |  |
| Title | 1 | Identify the report as a systematic review, meta-analysis, or both. | Page 1 |
| **ABSTRACT** | | |  |
| Abstract | 2 | See the PRISMA 2020 for Abstracts checklist. | Page 1-2 |
| **INTRODUCTION** | | |  |
| Rationale | 3 | Describe the rationale for the review in the context of existing knowledge. | Page 2-5 |
| Objectives | 4 | Provide an explicit statement of the objective(s) or question(s) the review addresses. | Page 2-5 |
| **METHODS** | | |  |
| Eligibility criteria | 5 | Specify the inclusion and exclusion criteria for the review and how studies were grouped for the syntheses. | Page 5-6 |
| Information sources | 6 | Specify all databases, registers, websites, organisations, reference lists and other sources searched or consulted to identify studies. Specify the date when each source was last searched or consulted. | Page 5 |
| Search strategy | 7 | Present the full search strategies for all databases, registers and websites, including any filters and limits used. | Page 5, S1 Table |
| Selection process | 8 | Specify the methods used to decide whether a study met the inclusion criteria of the review, including how many reviewers screened each record and each report retrieved, whether they worked independently, and if applicable, details of automation tools used in the process. | Page 5-6 |
| Data collection process | 9 | Specify the methods used to collect data from reports, including how many reviewers collected data from each report, whether they worked independently, any processes for obtaining or confirming data from study investigators, and if applicable, details of automation tools used in the process. | Page 6 |
| Data items | 10a | List and define all outcomes for which data were sought. Specify whether all results that were compatible with each outcome domain in each study were sought (e.g. for all measures, time points, analyses), and if not, the methods used to decide which results to collect. | Page 5-7 |
|  | 10b | List and define all other variables for which data were sought (e.g. participant and intervention characteristics, funding sources). Describe any assumptions made about any missing or unclear information. | Page 7-8 |
| Study risk of bias assessment | 11 | Specify the methods used to assess risk of bias in the included studies, including details of the tool(s) used, how many reviewers assessed each study and whether they worked independently, and if applicable, details of automation tools used in the process. | Page 7, S4 Table |
| Effect measures | 12 | Specify for each outcome the effect measure(s) (e.g. risk ratio, mean difference) used in the synthesis or presentation of results. | Page 7, S4 Table |
| Synthesis methods | 13a | Describe the processes used to decide which studies were eligible for each synthesis (e.g. tabulating the study intervention characteristics and comparing against the planned groups for each synthesis (item #5)). | Page 5-7 |
|  | 13b | Describe any methods required to prepare the data for presentation or synthesis, such as handling of missing summary statistics, or data conversions. | Page 6-8 |
|  | 13c | Describe any methods used to tabulate or visually display results of individual studies and syntheses. | Page 7-8, S3 Table |
|  | 13d | Describe any methods used to synthesize results and provide a rationale for the choice(s). If meta-analysis was performed, describe the model(s), method(s) to identify the presence and extent of statistical heterogeneity, and software package(s) used. | Page 7-8 |
|  | 13e | Describe any methods used to explore possible causes of heterogeneity among study results (e.g. subgroup analysis, meta-regression). | Page 8 |
|  | 13f | Describe any sensitivity analyses conducted to assess robustness of the synthesized results. | Page 8-9 |
| Reporting bias assessment | 14 | Describe any methods used to assess risk of bias due to missing results in a synthesis (arising from reporting biases). | Page 7-8, S4 Table |
| Certainty assessment | 15 | Describe any methods used to assess certainty (or confidence) in the body of evidence for an outcome. | Page 7-8 |
| **RESULTS** | | |  |
| Study selection | 16a | Describe the results of the search and selection process, from the number of records identified in the search to the number of studies included in the review, ideally using a flow diagram. | Page 5-6, Fig 1. |
|  | 16b | Cite studies that might appear to meet the inclusion criteria, but which were excluded, and explain why they were excluded. | Page 6-7, S1 File. |
| Study characteristics | 17 | Cite each included study and present its characteristics. | S3 Table |
| Risk of bias in studies | 18 | Present assessments of risk of bias for each included study. | Page 8-9, S4 Table |
| Results of individual studies | 19 | For all outcomes, present, for each study: (a) summary statistics for each group (where appropriate) and (b) an effect estimate and its precision (e.g. confidence/credible interval), ideally using structured tables or plots. | S4 Table |
| Results of syntheses | 20a | For each synthesis, briefly summarise the characteristics and risk of bias among contributing studies. | Page 9, S4Table |
|  | 20b | Present results of all statistical syntheses conducted. If meta-analysis was done, present for each the summary estimate and its precision (e.g. confidence/credible interval) and measures of statistical heterogeneity. If comparing groups, describe the direction of the effect. | Page 9-12 |
|  | 20c | Present results of all investigations of possible causes of heterogeneity among study results. | Page 12 |
|  | 20d | Present results of all sensitivity analyses conducted to assess the robustness of the synthesized results. | Page 12 |
| Reporting biases | 21 | Present assessments of risk of bias due to missing results (arising from reporting biases) for each synthesis assessed. | Page 12 |
| Certainty of evidence | 22 | Present assessments of certainty (or confidence) in the body of evidence for each outcome assessed. | Page 9, S4 Table |
| **DISCUSSION** | | |  |
| Discussion | 23a | Provide a general interpretation of the results in the context of other evidence. | Page 13-15 |
|  | 23b | Discuss any limitations of the evidence included in the review. | Page 16 |
|  | 23c | Discuss any limitations of the review processes used. | Page 16 |
|  | 23d | Discuss implications of the results for practice, policy, and future research. | Page 13-16 |
| **OTHER INFORMATION** | | |  |
| Registration and protocol | 24a | Provide registration information for the review, including register name and registration number, or state that the review was not registered. | NA |
|  | 24b | Indicate where the review protocol can be accessed, or state that a protocol was not prepared. | NA |
|  | 24c | Describe and explain any amendments to information provided at registration or in the protocol. | NA |
| Support | 25 | Describe sources of financial or non-financial support for the review, and the role of the funders or sponsors in the review. | Page 17 |
| Competing interests | 26 | Declare any competing interests of review authors. | NA |
| Availability of data, code and other materials | 27 | Report which of the following are publicly available and where they can be found: template data collection forms; data extracted from included studies; data used for all analyses; analytic code; any other materials used in the review. | Page 17 |

**S3 Table. Characteristics of included studies.**

| Author | Year | Country | Study type | Multi-centric | Data time | Setting | Only COVID-19 | Mode | Sample size | PP during ECMO | Main outcomes | Quality assessment |
| --- | --- | --- | --- | --- | --- | --- | --- | --- | --- | --- | --- | --- |
| Combes et al^34^ | 2018 | France, USA, Australia, Canada | RCT* | Yes | 2011.09-2017.11 | 64 centers participating in the EOLIA trial | No | VV | 124 | 70 | 60d survival | 7/NOS |
| Guervilly et al^23^ | 2019 | France | Retrospective, observational | No | 2012.01-2017.04 | Medical Intensive Care Unit North Hospital | No | VV | 168 | 91 | 1 month survival, 60d survival, 90d survival, ECMO weaning, ECMO duration, Length of ICU stay | 8/NOS |
| Giani et al^25^ | 2020 | Italy | Retrospective, observational | Yes | 2014.01-2018.12 | Six Italian ECMO referral centers | No | VV | 240 | 107 | Hospital discharge survival, 28d survival, ECMO duration, Length of ICU stay | 9/NOS |
| Rilinger et al^11^ | 2020 | Germany | Retrospective, observational | No | 2010.10-2018.05 | The Interdisciplinary Medical ICU at the Medical Centre | No | VV | 158 | 38 | Hospital discharge survival, 1 month survival, ECMO weaning, ECMO Duration, Length of ICU stay | 8/NOS |
| Garcia et al^31^ | 2020 | France | Retrospective, observational | No | unclear | Coronavirus Unit in Intensive Care at Lille University Central Hospital | Yes | VV | 25 | 14 | 1 month Survival, 28d survival, ECMO weaning, ECMO duration | 7/NOS |
| Yang et al^32^ | 2020 | China | Retrospective, observational | Yes | 2020.01-2020.05 | 21 ICU in Hubei, China | Yes | VV | 73 | 51 | 60d survival | 8/NOS |
| Chaplin et al^33^ | 2021 | New Zealand | Retrospective, observational | No | 2014.07-2019.07 | 26-bed quaternary cardiothoracic and vascular ICU (CVICU) in Auckland | No | VV | 72 | 13 | Hospital discharge survival, 90d survival, ECMO duration | 7/NOS |
| Laghlam et al^29^ | 2021 | France | Prospective, observational | No | 2020.03-2021.06 | 24-bed ICU of Cochin University Hospital | Yes | VV | 24 | 10 | 28d survival, 60d Survival, ECMO weaning, ECMO duration | 7/NOS |
| Lebreton et al^28^ | 2021 | France | Retrospective, observational | Yes | 2020.03-2020.06 | 17 Greater Paris ICU | Yes | VV, VA | 302 | 193 | 90d survival | 7/ NOS |
| Zaaqoq et al^30^ | 2022 | Australia | Retrospective, observational | Yes | 2020.02-2020.10 | Coronavirus Disease 2019 Critical Care Consortium international registry | Yes | VV | 232 | 67 | Hospital discharge survival | 8/NOS |
| Chen et al^22^ | 2022 | China | Retrospective, observational | No | 2013.04-2020.10 | 30-bed medical ICU | No | VV | 91 | 38 | Hospital survival, ECMO weaning, Length of ICU stay, ECMO duration | 8/NOS |
| Petit et al^24^ | 2022 | France | Retrospective, observational | No | 2012.01-2020.01 | 26-bed ICU in a tertiary center | No | VV | 298 | 64 | 90d survival, ECMO duration, Length of ICU stay | 8/NOS |
| Massart et al^26^ | 2023 | France | Retrospective, observational | Yes | Before 2021.06 | 47 centers, academic or nonacademic in France | Yes | VV | 517 | 364 | Hospital discharge survival, Length of ICU stay; ECMO duration | 9/NOS |
| Binda et al^36^ | 2023 | Italy | Cross-sectional | No | 2015.01-2019.12 | A tertiary level intensive care unit (ICU) in Milan (Italy) | No | VV | 114 | 62 | ECMO duration, Length of ICU stay | Moderate quality |
| Schmidt et al^12^ | 2023 | France | RCT | Yes | 2021.03-2021.12 | 14 ICUs in France | No | VV | 170 | 86 | 60d survival,90d survival, ECMO duration, ECMO weaning, Length of ICU stay | High risk of bias |

Et al: et alia.‌

RCT: randomized controlled trial. RCT*: randomized controlled trial comparing ECMO and controls with ECMO as a late rescue strategy.

NOS, Newcastle-Ottawa scale (the highest score of NOS is 9).

COVID-19: Corona Virus Disease 2019.

ECMO: extracorporeal membrane oxygenation. PP: prone position.

VV: Venovenous extracorporeal membrane oxygenation; VA: Venoarterialvenous extracorporeal membrane oxygenation.

**S4a Table. Quality assessment according to Newcastle-Ottawa quality assessment scale for cohort studies.**

| Study | Representativeness of the exposed cohort | Selection of the non-exposed cohort | Ascertainment of exposure | Demonstration that outcome of interest was not present at start of study | Comparability of cohorts on the basis of the design or analysis controlled for confounders | Assessment of outcome | Was follow-up long enough for outcomes to occur | Adequacy of follow up of cohorts | Total score (out of 9) | Description |
| --- | --- | --- | --- | --- | --- | --- | --- | --- | --- | --- |
| Chaplin2021 | 0 | 1 | 1 | 1 | 1 | 1 | 1 | 1 | 7 | Single-center study, confounding control is not clearly stated |
| Chen2021 | 0 | 1 | 1 | 1 | 2 | 1 | 1 | 1 | 8 | Single-center study |
| Combes2018 | 1 | 1 | 1 | 1 | 0 | 1 | 1 | 1 | 7 | Confounding variables were not controlled, and comparability between groups was poor |
| Garcia2021 | 0 | 1 | 1 | 1 | 1 | 1 | 1 | 1 | 7 | Single-center study, confounding control is not clearly stated |
| Giani2021 | 1 | 1 | 1 | 1 | 2 | 1 | 1 | 1 | 9 | High quality |
| Guervilly2019 | 0 | 1 | 1 | 1 | 2 | 1 | 1 | 1 | 8 | Single-center study |
| Laghlam2021 | 0 | 1 | 1 | 1 | 1 | 1 | 1 | 1 | 7 | Single-center study |
| Lebreton2021 | 1 | 1 | 1 | 1 | 0 | 1 | 1 | 1 | 7 | Confounding variables were not controlled, and comparability between groups was poor |
| Massart2023 | 1 | 1 | 1 | 1 | 2 | 1 | 1 | 1 | 9 | High quality |
| Petit2022 | 0 | 1 | 1 | 1 | 2 | 1 | 1 | 1 | 8 | Single-center study |
| Rilinger2020 | 0 | 1 | 1 | 1 | 2 | 1 | 1 | 1 | 8 | Single-center study |
| Yang2020 | 1 | 1 | 1 | 1 | 1 | 1 | 1 | 1 | 8 | Confounding variables were not controlled |
| Zaaqoq2022 | 1 | 1 | 1 | 1 | 1 | 1 | 1 | 1 | 8 | Confounding variables were not controlled |

**S4b Table.** **the Cochrane RoB2 tool for randomized controlled trial.**

| Study | Item | Risk of bias | Description |
| --- | --- | --- | --- |
| Schmidt2023 | Randomization process | Low | Randomization was performed through a secure web-based system using the minimization method. |
|  | Deviations from intended interventions | High | Due to the nature of the intervention (prone versus supine), blinded testing of patients and staff was not possible. No information was provided on whether the outcome assessors were blind. |
|  | Missing outcome data | Low | All randomized patients completed the study, and none were lost to follow-up. |
|  | Measurement of the outcome | Low | This study followed the CONSORT reporting guidelines and were registered on the clinicalTrials.gov to help ensure the completeness of report. |
|  | Selection of the reported result | Some concern | The study does not provide enough information to assess whether it is biased. |
|  | Overall risk of bias | High risk of bias | |

**S4c Table. Quality assessment according to** **Agency for Healthcare Research and Quality assessment scale for cross-sectional study.**

| Study | Items | Yes/No/Unclear | Description |
| --- | --- | --- | --- |
| Binda2023 | 1. Define the source of information (survey, record review) | Yes | The study was conducted in a tertiary ICU in Milan, Italy, from January 2015 to December 2019 |
|  | 2. List inclusion and exclusion criteria for exposed and unexposed subjects (cases and controls) or refer to previous publications | Yes | Detailed inclusion and exclusion criteria are listed in the article |
|  | 3. Indicate time period used for identifying patients | Yes | From January 2015 to December 2019 |
|  | 4. Indicate whether or not subjects were consecutive if not population-based | Yes | The subjects are enrolled consecutively |
|  | 5. Indicate if evaluators of subjective components of study were masked to other aspects of the status of the participants | No | Not mentioned |
|  | 6. Describe any assessments undertaken for quality assurance purposes (e.g., test/retest of primary outcome measurements | No | Not mentioned |
|  | 7. Explain any patient exclusions from analysis | No | Not mentioned |
|  | 8. Describe how confounding was assessed and/or controlled. | Yes | Use a logical model and consider multiple variables |
|  | 9. If applicable, explain how missing data were handled in the analysis | No | Not mentioned |
|  | 10. Summarize patient response rates and completeness of data collection | No | Not mentioned |
|  | 11. Clarify what follow-up, if any, was expected and the percentage of patients for which incomplete data or follow-up was obtained | No | Not mentioned |

**S4d Table. GRADE assess the overall quality of evidence.**

| **Certainty assessment** | | | | | | | **№ of patients** | | **Effect** | | **Certainty** |
| --- | --- | --- | --- | --- | --- | --- | --- | --- | --- | --- | --- |
| **№ of studies** | **Study design** | **Risk of bias** | **Inconsistency** | **Indirectness** | **Imprecision** | **Other considerations** | **ECMO+PP** | **ECMO only** | **Relative (95% CI)** | **Absolute (95% CI)** |  |
| **hospital discharge survival** | | | | | | | | | | | |
| 6 | non-randomized studies | serious | not serious | not serious | serious | publication bias strongly suspected | 326/627 (52.0%) | 299/683 (43.8%) | **OR 1.26** (0.85 to 1.86) | **57 more per 1,000** (from 40 fewer to 154 more) | ⨁◯◯◯ Very low |
| **1 month survival** | | | | | | | | | | | |
| 5 | non-randomized studies | serious | not serious | not serious | serious | publication bias strongly suspected | 104/166 (62.7%) | 140/281 (49.8%) | **OR 1.20** (0.46 to 3.14) | **45 more per 1,000** (from 185 fewer to 259 more) | ⨁◯◯◯ Very low |
| **60d survival** | | | | | | | | | | | |
| 5 | non-randomized studies | not serious | not serious | not serious | serious | publication bias strongly suspected | 129/251 (51.4%) | 132/256 (51.6%) | **OR 1.36** (0.78 to 2.37) | **76 more per 1,000** (from 62 fewer to 201 more) | ⨁◯◯◯ Very low |
| **90d survival** | | | | | | | | | | | |
| 5 | non-randomized studies | not serious | not serious | not serious | serious | publication bias strongly suspected | 250/447 (55.9%) | 278/563 (49.4%) | **OR 1.68** (1.00 to 2.80) | **127 more per 1,000** (from 0 fewer to 238 more) | ⨁◯◯◯ Very low |
| **ECMO Duration** | | | | | | | | | | | |
| 11 | non-randomized studies | not serious | not serious | not serious | not serious | none | 887 | 990 | - | SMD **1.99 higher** (1.27 higher to 2.7 higher) | ⨁⨁◯◯ Low |
| **Length of ICU Stay** | | | | | | | | | | | |
| 9 | non-randomized studies | not serious | not serious | not serious | serious | none | 860 | 920 | - | SMD **1.17 higher** (0.58 higher to 1.75 higher) | ⨁◯◯◯ Very low |
| **ECMO Weaning** | | | | | | | | | | | |
| 6 | non-randomized studies | not serious | not serious | not serious | not serious | publication bias strongly suspected | 150/277 (54.2%) | 168/359 (46.8%) | **OR 1.17** (0.65 to 2.11) | **39 more per 1,000** (from 104 fewer to 182 more) | ⨁◯◯◯ Very low |

CI: confidence interval; OR: odds ratio; SMD: standardized mean difference

**S5 Table. Criteria used for the matching procedure.**

| Author | Guervilly et al. | Rilinger et al. | Giani et al. | Petit et al. | Chen et al. | Massart et al. |
| --- | --- | --- | --- | --- | --- | --- |
| Publication year | 2019 | 2020 | 2021 | 2021 | 2022 | 2023 |
| Matching technique | 5 criteria | Propensity score | Propensity score | Propensity score | Propensity score | Propensity score |
| Criteria used for matching |  |  |  |  |  |  |
| Age | Yes | Yes | Yes | Yes | Yes | Yes |
| Gender | Yes | Yes | Yes | No | Yes | Yes |
| SOFA score | Yes | Yes | Yes | Yes | No | No |
| Duration of MV before ECMO | Yes | Yes | Yes | Yes | Yes | Yes |
| Use of PP before ECMO | Yes | Yes | No | Yes | Yes | Yes |
| Others | No | No | BMI, hypertension, diabetes, immunodeficiency, other chronic diseases, use of renal replacement therapy before ECMO, cause of ARDS, PaO2:FiO2ratio | BMI, SAPS II score, immunodeficiency, corticosteroids use, static compliance, viral pneumonia, PaO2: FiO2ratio | BMI, Cause of ARDS, Comorbidities, Smoking, Alcohol, Fluid balance, PaO2/FiO2 ratio | BMI, Comorbidities, period of enrollment, Treatment before cannulation, Pao2/Fio2 ratio, Paco2, Lung compliance, Positive end-expiratory pressure, Tidal volume, Respiratory rate, other support at ECMO cannulation, Details of cannulation, Center experience |

Et al: et alia.‌
