# Supplementary material for: Effect of prone positioning in adult patients receiving veno-venous extracorporeal membrane oxygenation: A meta-analysis
Source: PLoS One. 2025 Mar 25;20(3):e0320532. doi: 10.1371/journal.pone.0320532 (PMC11936214; doi:10.1371/journal.pone.0320532)
Supplement: S3 File — S1 Fig. Forest plot of long-term survival. S2 Fig. Survival on day-60 for subgroups based on COVID-19 or not. S3 Fig. Survival on day-90 for subgroups based on COVID-19 or not. S4 Fig. Long-term survival after matching. S5 Fig. A ECMO duration. B ECMO weaning. C Length of ICU stay. S6 Fig. Publication bias was evaluated by Begg’s test: A hospital discharge survival, B 1-month survival, C 60-d survival, D 90-d survival, E ECMO duration, F ECMO weaning, G Length of ICU stay. S7 Fig. Publication bias was evaluated by Egger’s test: A hospital discharge survival, B 1-month survival, C 60-d survival, D 90-d survival, E ECMO duration, F ECMO weaning, G Length of ICU stay. S8 Fig. Publication bias was evaluated by funnel plot: A hospital discharge survival, B 1-month survival, C 60-d survival, D 90-d survival, E ECMO duration, F ECMO weaning, G Length of ICU stay. S9 Fig. Sensitivity analyses: A hospital discharge survival, B 1-month survival, C 60-d survival, D 90-d survival, E ECMO duration, F ECMO weaning, G Length of ICU stay. (DOCX) [file pone.0320532.s003.docx]

**
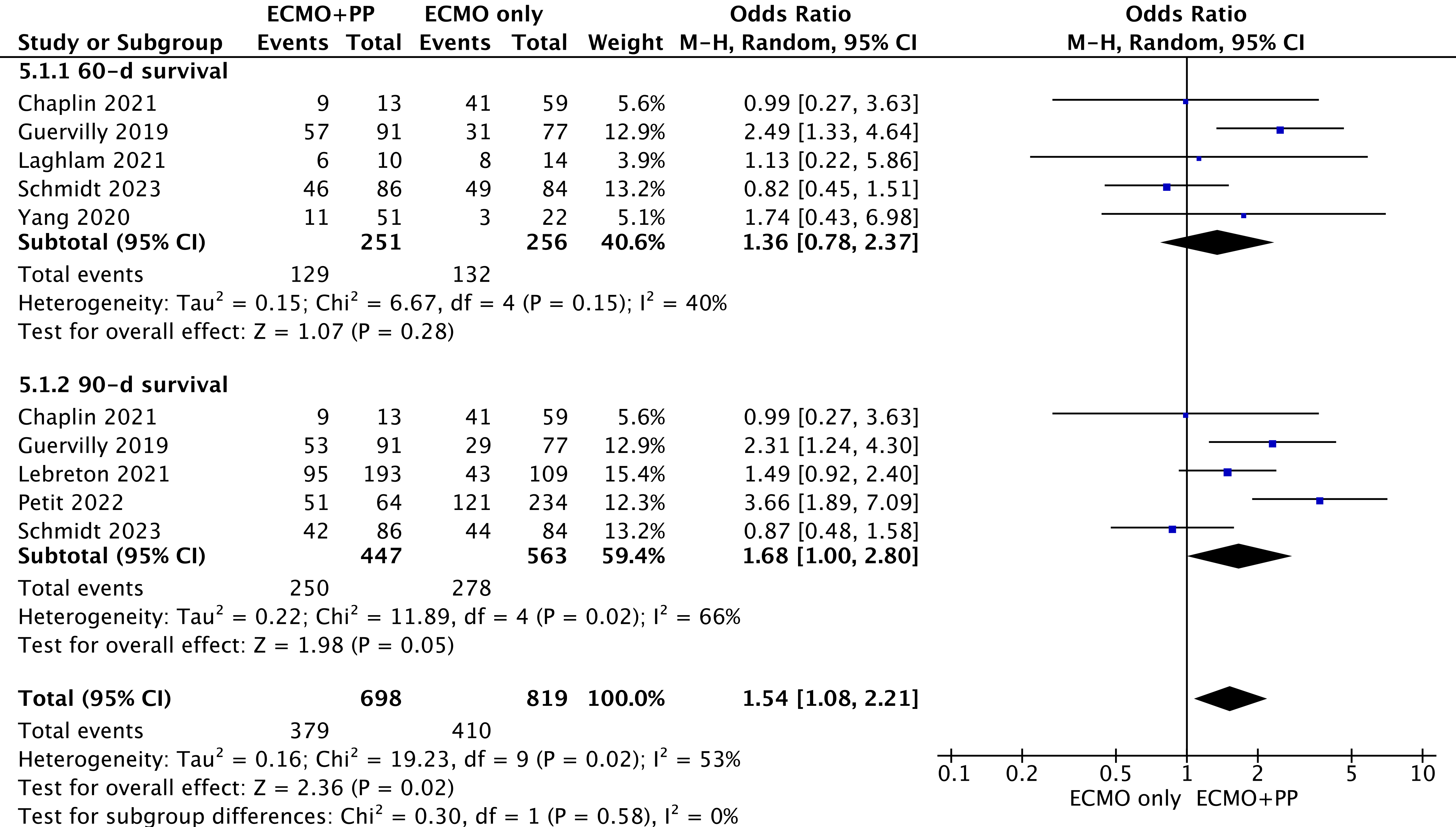
** **S1 Fig. Forest plot of long-term survival.**

**
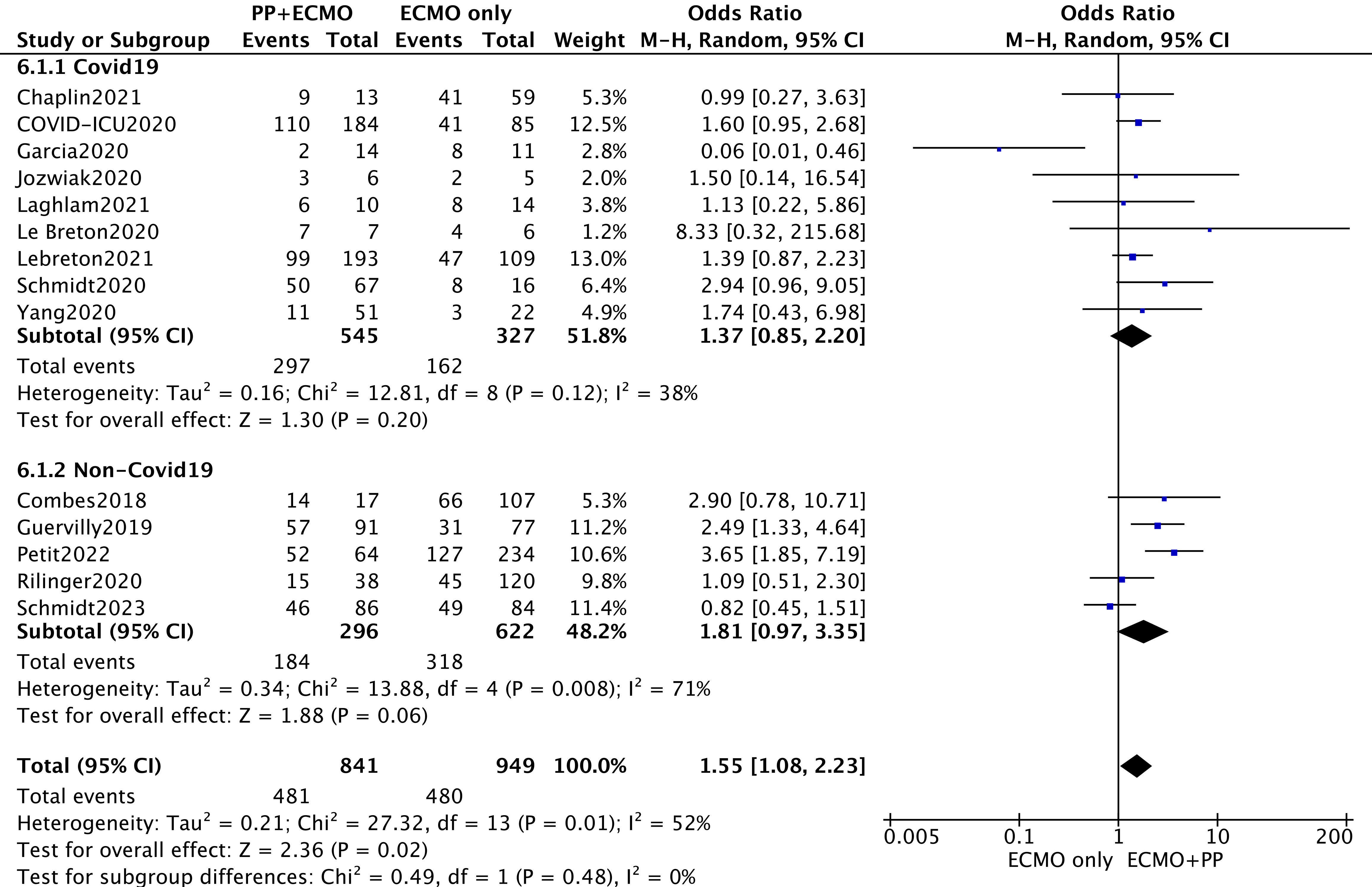
 S2 Fig. Survival on day-60 for subgroups based on COVID-19 or not.**


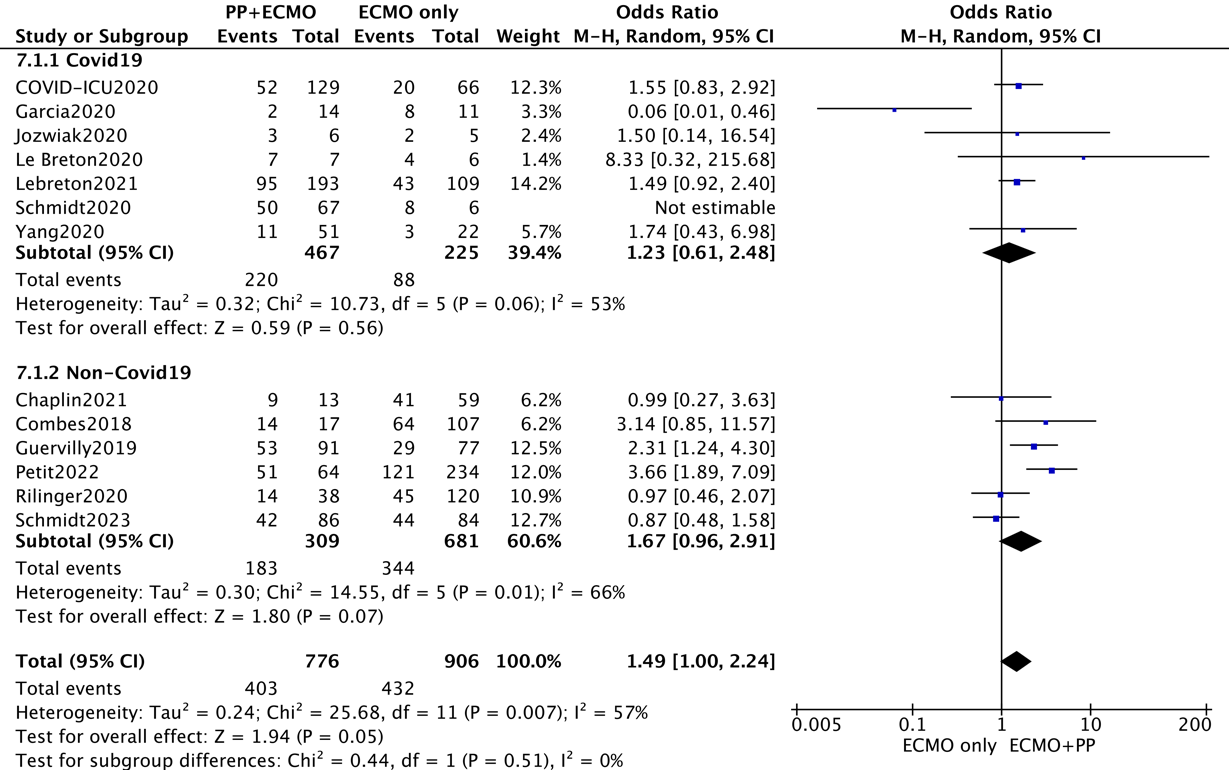
 **S3 Fig. Survival on day-90 for subgroups based on COVID-19 or not.**


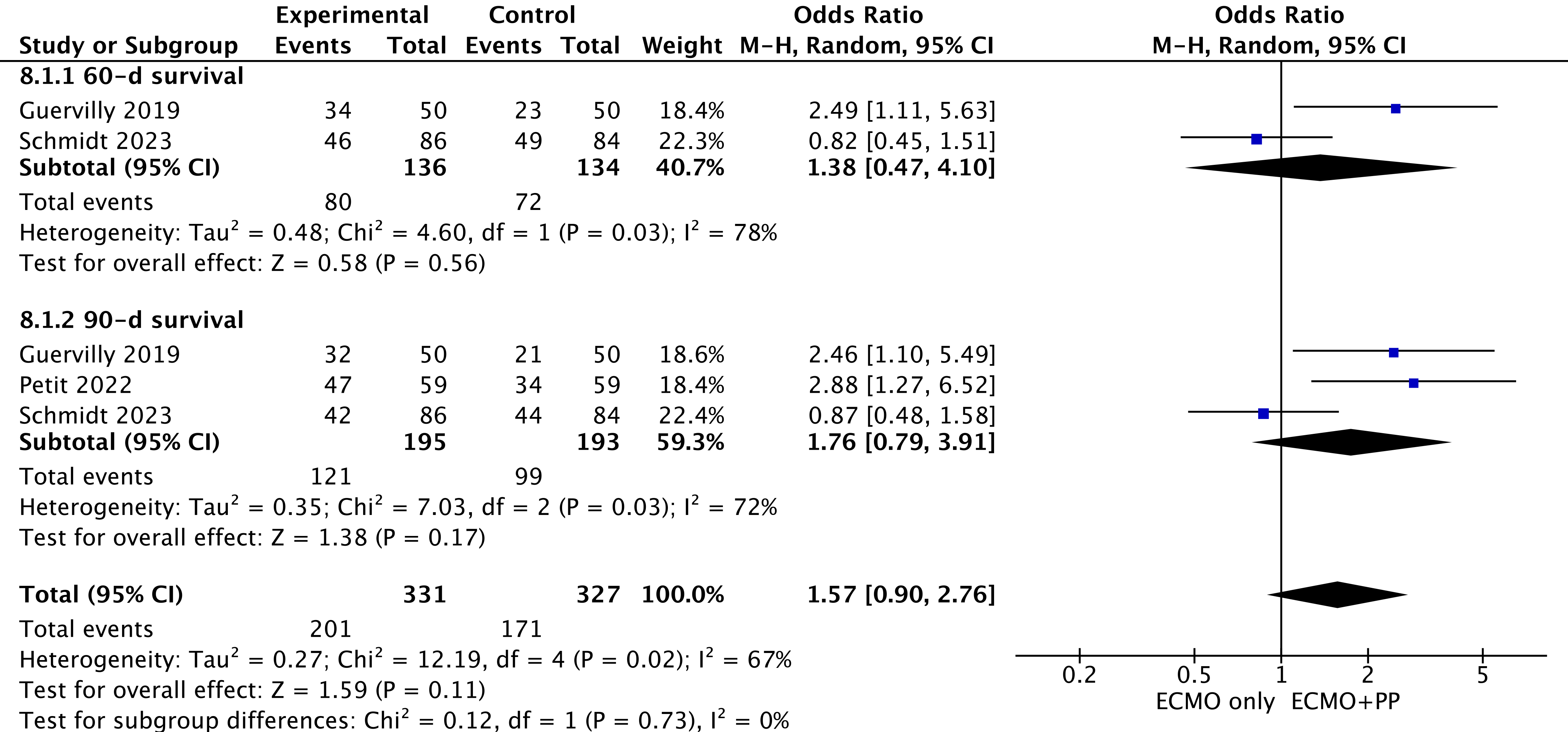


**S4 Fig. Long-term survival after matching.**

**
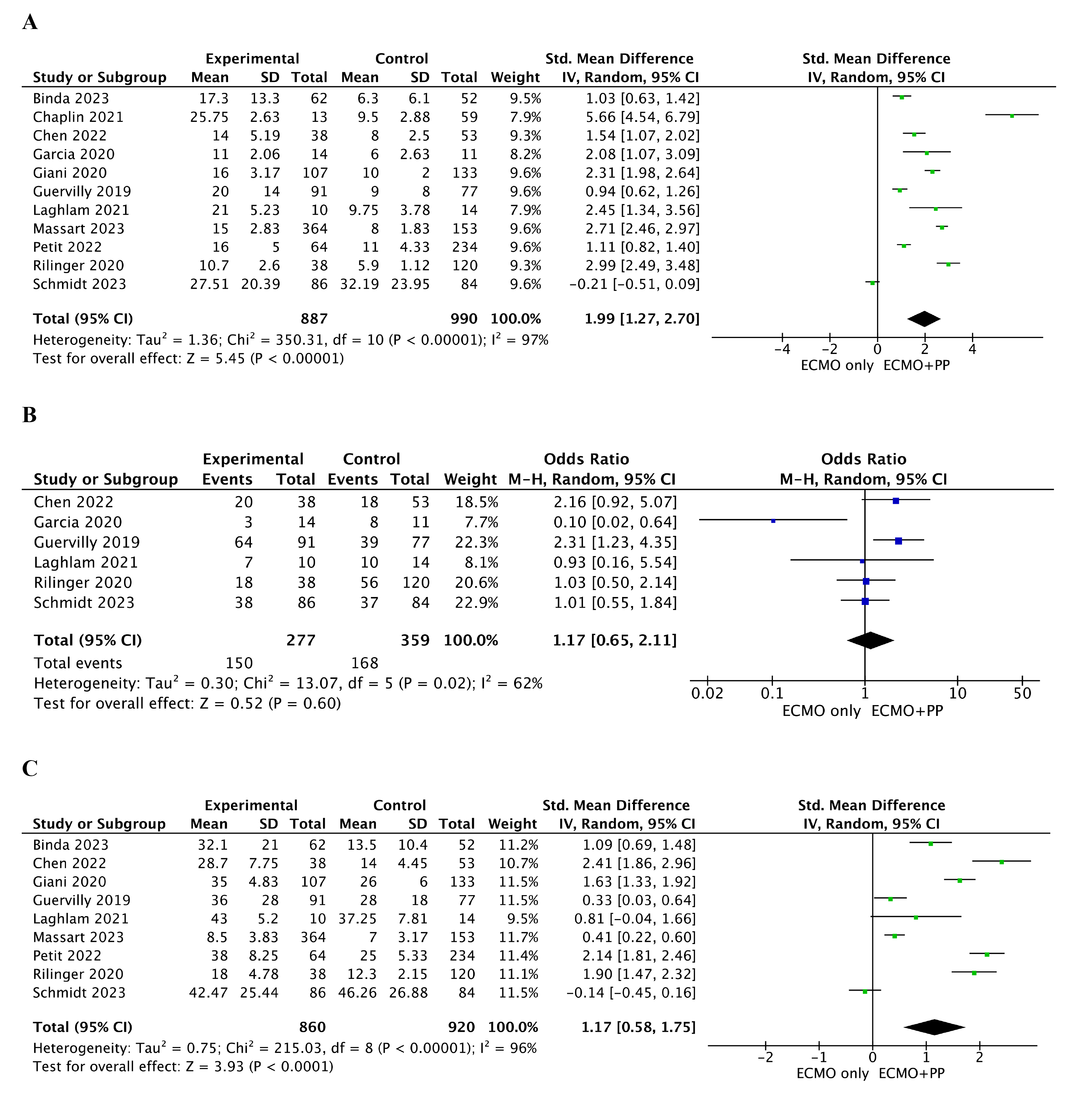
**

**S5 Fig. A ECMO duration. B ECMO weaning. C Length of ICU stay.**

**
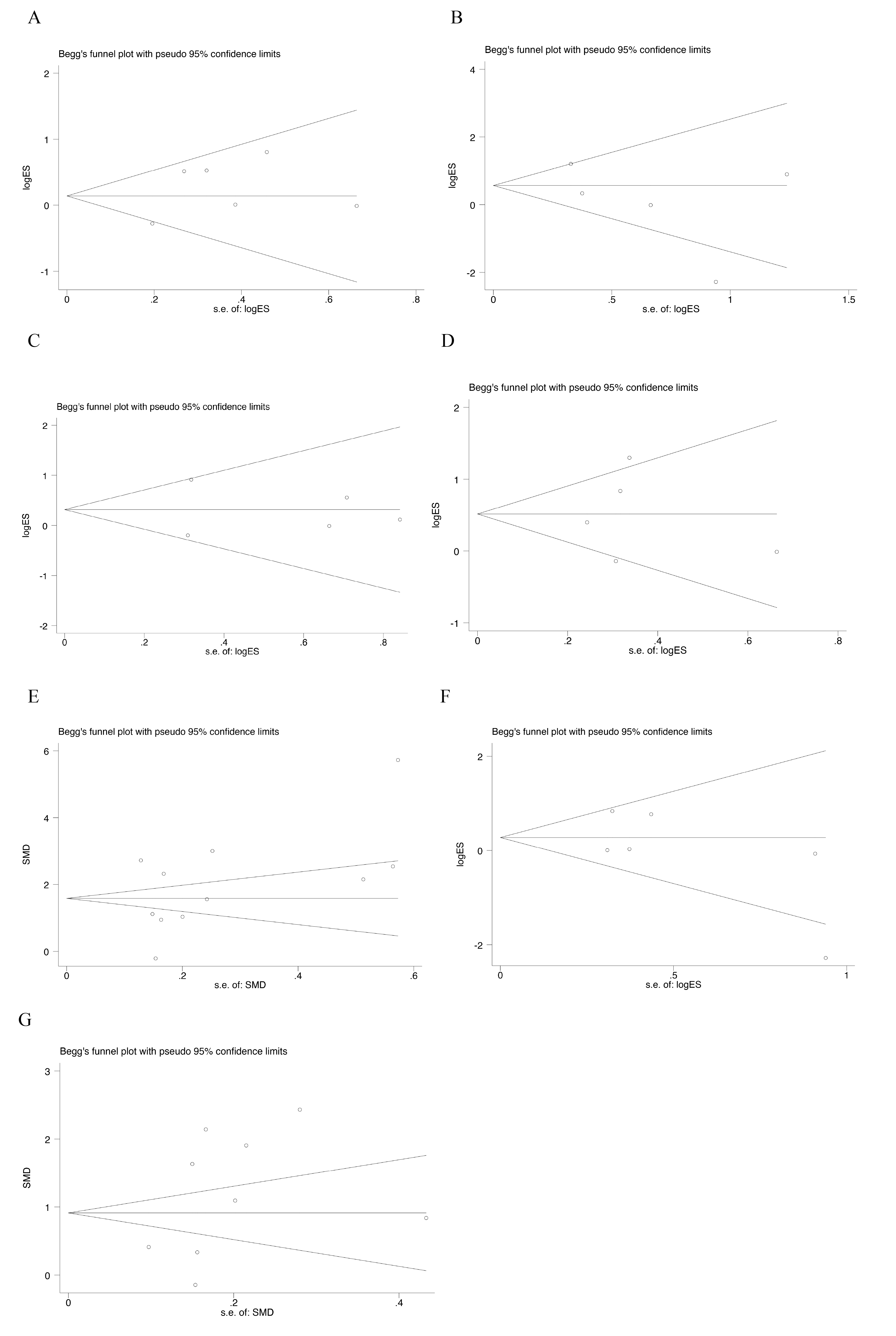
**

**S6 Fig. Publication bias was evaluated by Begg's test: A hospital discharge survival, B 1-month survival, C 60-d survival, D 90-d survival, E ECMO duration, F ECMO weaning, G Length of ICU stay.**

**
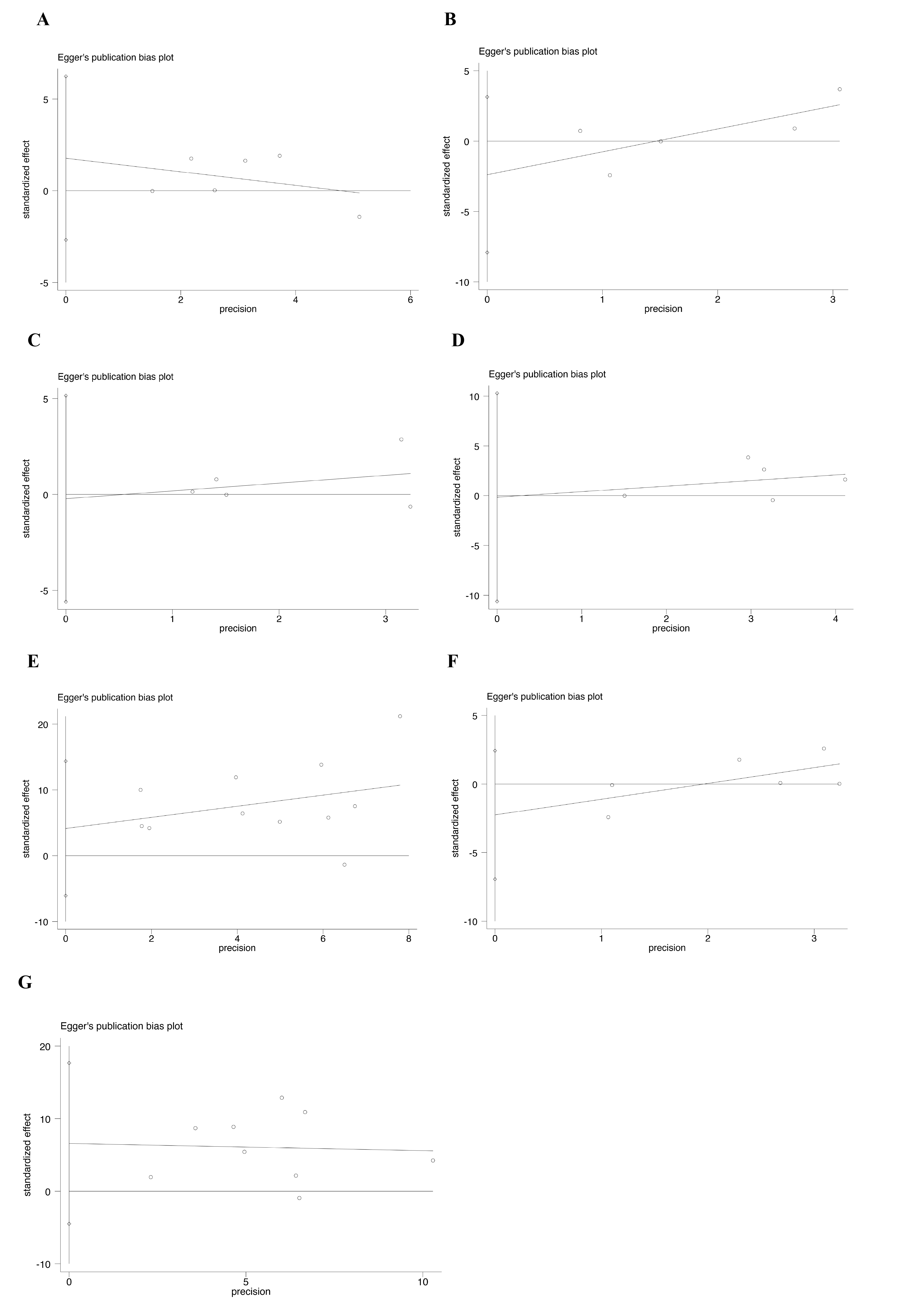
**

**S7 Fig. Publication bias was evaluated by Egger’s test: A hospital discharge survival, B 1-month survival, C 60-d survival, D 90-d survival, E ECMO duration, F ECMO weaning, G Length of ICU stay.**

**
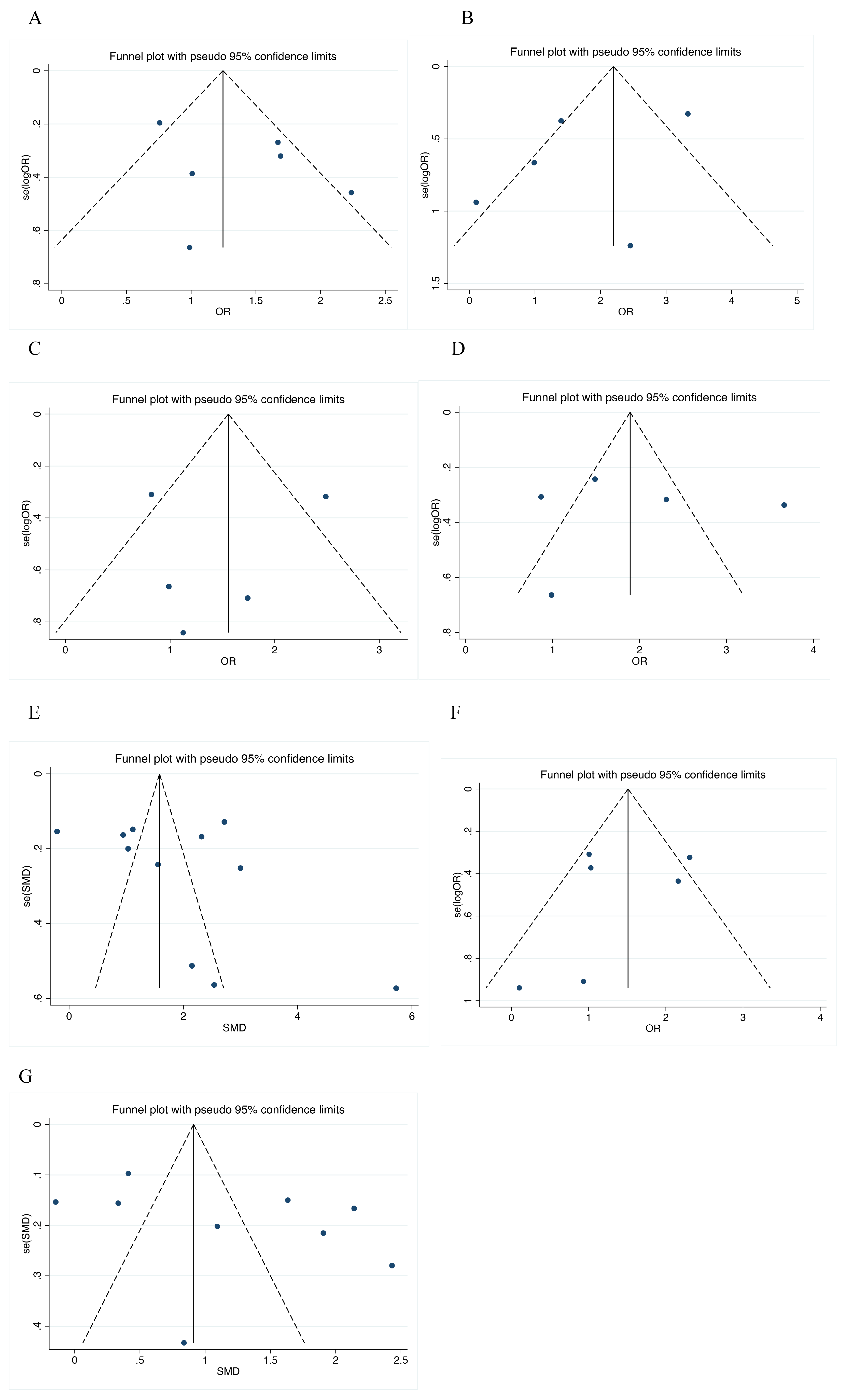
**

**S8 Fig. Publication bias was evaluated by funnel plot: A hospital discharge survival, B 1-month survival, C 60-d survival, D 90-d survival, E ECMO duration, F ECMO weaning, G Length of ICU stay.**

**
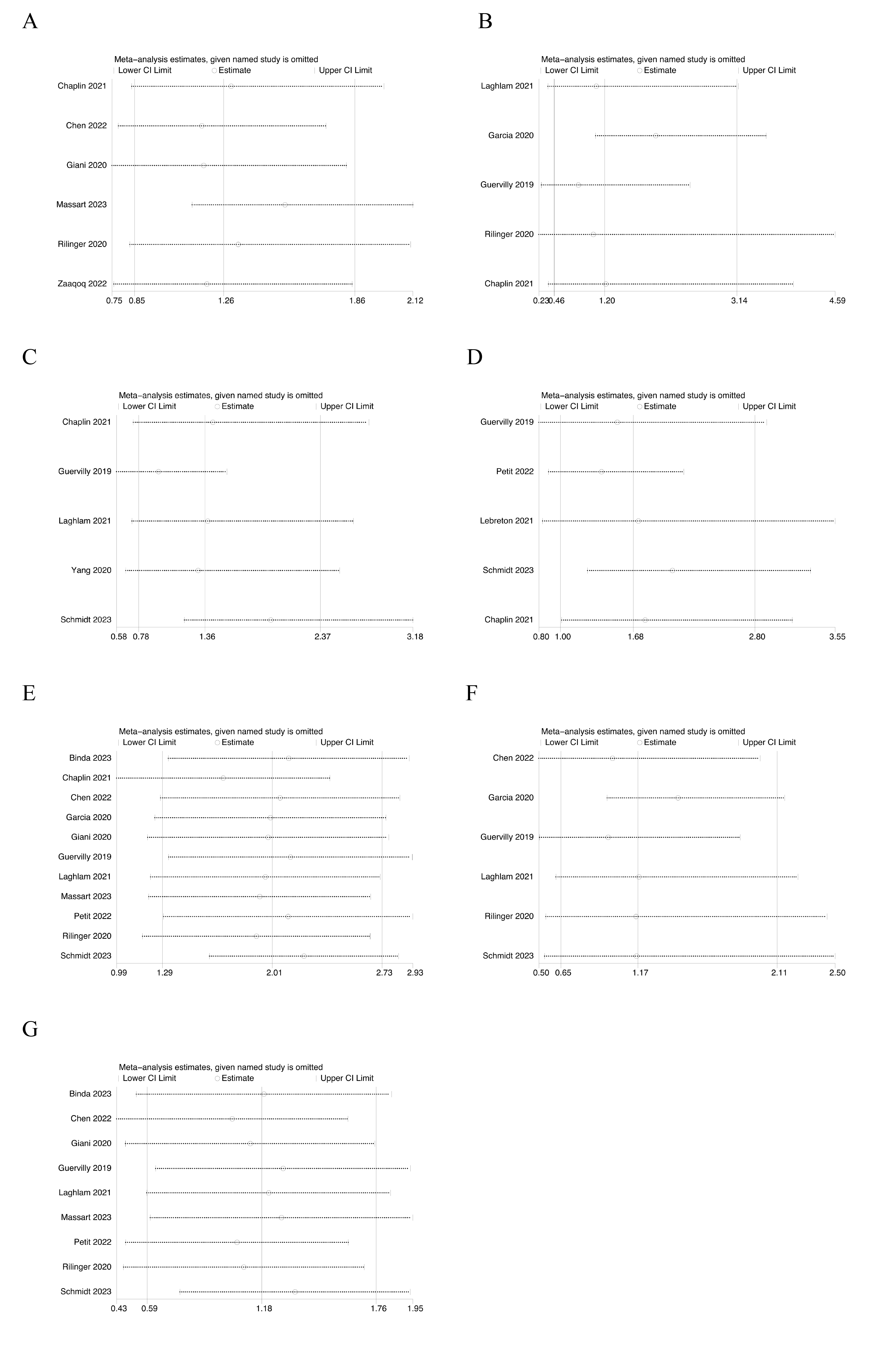
**

**S9 Fig. Sensitivity analyses: A hospital discharge survival, B 1-month survival, C 60-d survival, D 90-d survival, E ECMO duration, F ECMO weaning, G Length of ICU stay.**
